# Notes and News

**Published:** 1899-06-03

**Authors:** 


### Notes and News.

(Collected and Communicated.)

### HOSPITAL MEETINGS.

**Royal Orthopædic Hospital.—Special Court at half past four, on Thursday, June 8th, at 20, Hanover Square, W.**

**Friedenheim, Upper Avenue Road, N.W.—Annual Meeting, at half-past three, on Thursday, June 8th, in the Lecture Hall of the School for the Blind, adjoining "Friedenheim."**

**City of London Hospital for Diseases of the Chest.—Festival Dinner at half-past seven p.m., on June 7th, at Criterion Restaurant. Hon. Walter Rothschild in the chair.**

Mr. G. A. Hawkins-Ambler, F.R.C.S.E., has been appointed hon. assistant surgeon to the Liverpool Stanley Hospital.

By the visit of the Duke and Duchess of York to Lynn, the West Norfolk and Lynn Hospital has benefited to the extent of £1,188. No less than 121 purses were presented to the Duchess towards defraying the expenses of the new Jubilee wing.

Two cases of plague have occurred at Alexandria, and the port is now declared infected. Some time ago the Egyptian Government was approached with the object of abolishing the pilgrimages to Mecca, but no action was taken on religious grounds.

At the last quarterly court of the Middlesex Hospital it was announced that the new cancer wing will shortly be opened, and that arrangements are about to be made for the reception of tuberculous patients at the convalescent home at Clacton-on-Sea.

A sad accident happened to an ambulance lately at Liverpool. A patient was being conveyed to a hospital when the horse bolted, the ambulance was smashed, and the driver and the patient were both killed.

The medical staff of the North Adam's Hospital in Massachusetts have taken a vigorous line in regard to hospital abuse, having refused to treat patients at their hospital without payment unless they are actually fit objects of charity. But who is to decide? This action is the outcome of abuse of hospital treatment such as is frequently experienced in our own hospitals, but we may fairly doubt the applicability of the remedy to English conditions.

At Woodford a Jubilee hospital has been erected by Mr. J. R. Roberts, and this has been opened by the Duke and Duchess of Connaught.

The plague at Bombay is now rapidly decreasing. One or two more patients have been added to the list at Alexandria. Cholera is raging at Karachi, India, the death-roll being very large.

Mr. Maccur, Ex-Chief Magistrate of Dundee, has offered £10,000 to that city for the erection of a sanatorium for consumptives, and an anonymous donor has promised £500 for three years provided the sanatorium is established forthwith. The two gifts have been gratefully accepted, and the scheme will be started forthwith.

The efficacy of the fresh-air treatment is now so generally believed in that no institution for the treatment of tuberculosis can afford to ignore it. Even in London, at the Brompton Hospital for Consumption, arrangements to carry it out are to be made on the south side of the building. During a large portion of the year London air is by no means unhealthy, and, smuts ignored, many patients will be able to benefit by the outdoor life now available.

The Tuberculosis Congress which has been held at Berlin commenced with addresses on the origin and propagation of tuberculosis, and telegrams from various European Sovereigns in sympathy with the aims of the Congress were read. Dr. Fraenkel, speaking on the question of the infectiveness of tuberculosis, showed that, although the bacillus can live amid various surroundings, it really thrives only in the human body, and therefore is only to be found, as a rule, in the immediate neighbourhood of diseased persons. Again, he said that the human body is only in a very limited degree susceptible to infection, for it requires a repeated absorption of large quantities of the bacillus to set up the disease; hence the infection of tuberculosis comes into play almost wholly within families or among people who live crowded together in ill-ventilated work-rooms or sleeping-rooms.

The sixty-seventh session of the General Medical Council was opened last Tuesday. The president, Sir William Turner, in his opening address, touched on questions which were likely to arise: the amendment of the law as to death certification; the suspension of the right to use medical titles by persons whose names have been erased from the "Medical Register" for criminal or professional offences; the amendment of the law to restrain companies from conducting the practice of medicine, surgery, dentistry, and midwifery; and the preliminary education of medical students; and he referred to the cases of discipline which would come before the Council. Among the latter is the curious question, raised for the first time, whether a registered medical practitioner, the salaried officer of a provident dispensary, was to be regarded as covering an unqualified person who not only acted as dispenser, but, it was alleged, visited and prescribed for the patients attending the dispensary, the said dispenser not being engaged by the practitioner, but being in the employ of the committee, and the medical officer affirming that he had given no consent to the dispenser visiting his patients.

